# PUM2 Promotes Glioblastoma Cell Proliferation and Migration via Repressing BTG1 Expression

**DOI:** 10.1247/csf.18030

**Published:** 2019-02-16

**Authors:** Yuanyu Wang, Weili Sun, Jiankai Yang, Liang Yang, Chen Li, Hongjiang Liu, Xiaopeng Liu, Baohua Jiao

**Affiliations:** 1 Department of Neurosurgery, the Second Hospital of Hebei Medical University, No. 215 Hepingxi Road, Shijiazhuang City 050000, Hebei Province, China

**Keywords:** PUM2, BTG1, glioblastoma, cell proliferation, metastasis

## Abstract

PUM2, an RNA binding protein, is known to promote stem cell proliferation via repressing expressions of cell cycle genes. Similar with stem cells, malignant cells are characterized by unlimited proliferation and remote migration. However, roles of PUM2 in cancer development are controversial. Here, we investigated PUM2’s role in glioblastoma development and its relationship with the cell cycle regulator BTG1. Immunoblotting and RT-qPCR were used to evaluate protein expression level and transcript level, respectively. ShRNAs were designed to knock down PUM2 and BTG1 expression. CCK-8 assay was used to evaluate cell viability. Cell migration assay and evasion assay were used to evaluate metastatic capability of glioblastoma cell. RNA pull-down assay and RNA immunoprecipitation assay were used to test the interaction between PUM2 and BTG1 3’UTR. PUM2 expression is elevated in glioblastoma tumor tissues as well as glioblastoma cell lines. PUM2 knockdown remarkably suppresses glioblastoma cell proliferation and migration. In addition, PUM2 knockdown increases BTG1 expression. RNA pull-down assay and RNA immunoprecipitation assay show PUM2 binds to BTG1 3’UTR directly. Furthermore, knockdown of BTG1 reverses the effect of PUM2 knockdown on glioblastoma cell proliferation and migration. Our results suggest that PUM2 promote glioblastoma development via repressing BTG1 expression.

## Introduction

Glioblastoma is the most common CNS primary malignancy in adults with a median survival of 15 months (Omuro and DeAngelis, 2013). Maximal resection followed by temozolomide (TMZ) and radiation is currently the most optimized treatment regimen that a clinician can use to treat newly diagnosed patients (Karachi *et al.*, 2018; Lacroix *et al.*, 2001). Even with such a regimen, the survival median remains short at between 15 and 18 months with only 10% 5-year survivors (Mortazavi, 2018; Woo *et al.*, 2018). Though the biological behaviors of glioblastoma are characterized by rapid proliferation and aggressive local invasion in CNS, the molecular genetics of glioblastoma remains poorly understood, which impedes the development of more effective treatments. Based on the behavioral characteristics of glioblastoma, most efforts to identify the molecular targets that mediate the sensitivity and resistance to treatment focus on cell cycle checkpoint genes as well as growth factor receptors (Monticelli *et al.*, 2018). However, roles of these genes in the etiology of glioblastoma remain unsettled. Here, we focus on the positive cell cycle regulator PUM2 and its potential target BTG1 which inhibits cancer cell proliferation and promotes apoptosis.

PUM2 is a RNA binding protein and is known to functions as a translational repressor by binding to the 3’UTR region of mRNAs (Fox *et al.*, 2005; White *et al.*, 2001). More than 1,000 distinct mRNAs have been identified to carry the core PUM2 binding motif, suggesting PUM2 is a broad posttranscriptional regulator of many genes that are involved in a variety of cellular pathways such as cell proliferation and differentiation (Galgano *et al.*, 2008). One previous study has shown that knockout of PUM2 in CNS dramatically reduced the number of neural stem cells and subsequently inhibited neurogenesis. In the same study, PUM2 was shown to target 3’UTR region of hundreds of genes that are involved in cell adhesion, cell migration, synapse function, neuron differentiation, and development (Zhang *et al.*, 2017). These findings suggest that PUM2 promote neural stem cell proliferation and modulate neurogenesis by targeting multiple cellular pathways. In addition to positively regulate stem cell proliferation, emerging evidences suggest that PUM2 is implicated in the pathogenesis of some cancers including leukemia (Kawasaki *et al.*, 2018; Lee *et al.*, 2016; Naudin *et al.*, 2017). However, these findings are inconclusive regarding the roles of PUM2 in the pathogenesis of cancers. Furthermore, the exact cellular pathways targeted by PUM2 in cancer cells remain unexplored. In this study, we demonstrate that PUM2 positively promotes glioblastoma cell proliferation and migration mainly by repressing the expression of tumor suppressor gene BTG1.

## Methods and Materials

### Cell culture, lentiviral transduction and stable cell line establishment

Four glioblastoma cell lines U-87MG, U-T98G, U-251MG and A172 and two human astrocyte cell lines HA and NHA were obtained from the American Type Culture Collection. All cells were cultured in DMEM medium with glutamine, antibiotics and 10% FBS. Lentiviruses carrying shPUM2 (#a or #b), shBTG1, or control shRNA were packaged in HEK-293 T cells by using lentiviral packaging kit (Open Biosystems, AL, USA). Twenty-four hours after transduction, cells were treated with puromycin at 0.2 μg/ml for 72 hours and thereafter maintained in the medium with puromycin at 0.1 μg/ml.

### Immunohistochemistry and western blotting

Immunohistochemistry was performed as described in (Jeong *et al.*, 2017). Rabbit anti-human PUM2 polyclonal antibody was used at 1:100 dilution (Abcam, Cambridge, UK; ab10361). Tumor tissues and adjacent normal tissues were sampled by an experienced pathologist. The quality of immunohistochemical staining was evaluated by the same pathologist.

Western blotting was performed as described in (Yao *et al.*, 2015). RIPA lysis buffer (Thermo Fisher, USA; Cat#89900) was used to treat tissues and cultured cells. Rabbit anti-human PUM2 polyclonal antibody was used at 1:200 dilution (Abcam, Cambridge, UK; ab10361) and mouse anti-human BTG1 monoclonal antibody was used at 1:200 dilution (Abcam, Cambridge, UK; ab50991). Goat secondary antibody conjugated with horseradish peroxidase was used at 1:5000 dilution (P0008, Promoter, Wuhan, China). Imaging was done using an ECL-based system (Santa Cruz Biotechnol., CA, USA). Beta-actin was used as a loading control.

### Cell proliferation assay and cell viability assay

Stable glioblastoma cells transfected with PUM2 shRNA or control shRNA were seeded at 1.5×10^5^/well into 6-well plates. Within a period of 5 days after seeding, cells were suspended and counted every 24 hours by using the Z1 particle counter (Beckman Coulter, Inc., Brea, CA).

Stable glioblastoma cells transfected with PUM2 shRNA or control shRNA were seeded at 1,000/well into 96-well plates. Seventy-two hours after seeding, cells viability was evaluated by using the Cell-Counting Kit 8 (CCK8; Dojindo Laboratories).

### Transwell migration and invasion assay

Transwell chambers (6.5 mm wide) with a pore size of 8 μm were used to evaluate the capability of Cell migration. Chamber with matrigel 24-well DI kit (BD) was used to evaluate the capability of cell invasion. The assays were performed as described in (Wu *et al.*, 2014). Specifically, stable glioblastoma cells expressing PUM2 shRNA or control shRNA were suspended in serum-free medium and seeded into the upper chamber at 1×10^5^/well. The lower chamber was filled with DMEM medium. Twelve hours after incubation, 4% paraformaldehyde was used to fix the migrated/invaded cells in the lower chamber for 30 minutes. Cell death was not significant in each experimental group, which was confirmed by the comparable cell numbers remaining on the dishes. Crystal violet solution (0.1 mg/mL) was then used to stain the cells. The migrated/invaded cells were counted at 20× magnification under a dissecting microscope. For each preparation, six visual fields were counted, and the average was calculated. All experiments were performed in triplicate.

### Annexin V/PI staining and flow cytometry assay

FITC Annexin V apoptosis detection kid with PI (BioLegend, San Diego, CA) was used for apoptosis assay by flow cytometry following the instruction book. Briefly, cells were harvested and incubated with 5 mM EDTA (pH 7.4) for 15 minutes on ice. Appropriate amount of fluorescently conjugated annexin V and PI was added to stain for 15–20 minutes on ice. Proceed to flow cytometric measurement of the PI and annexin fluorescence. Data were collected and analyzed using Bioconductor 2.2.

### RNA extraction and qRT-PCR

RNA was extracted using TRIzol (Life Technologies, Grand Island, NY) from cultured cells or human tissues and reversely transcribed using the PrimeScript RT Reagent Kit (Takara, Japan). To evaluate the stability of BTG1 mRNA, cells seeded in 6-well plate were treated with actinomycin and RNA was extracted at hour 0, 2, 4, 6, 8. The transcriptional levels of PUM2 and BTG1 were normalized to those of the housekeeping gene GAPDH and fold changes were determined by relative quantification (2^–ΔΔCt^). The primers used for qRT–PCR were:

PUM2 sense, 5’-TTCTCAGCAGGCCTTGCTC-3’;

PUM2 antisense, 5’-GGTGGAACCACTGCTGGAC3’;

BTG1 sense, 5’-CACCATGATAGGCGAGATCG-3’;

BTG1 antisense, 5’-GCTGTCTACCATTTGCACG-3’;

GAPDH sense, 5’-CCACCCATGGCAAATTCCATGGCA-3’;

GAPDH antisense, 5’-TCTAGACGGCAGGTCAGGTCCACC-3’.

### Luciferase reporter assay

The 3’-UTR region of BTG1 was amplified using PCR from genomic DNA and cloned into the psiCHECK2 vector (Promega, Madison, WI, USA) between NotI and XhoI sites immediately downstream of the luciferase gene. Empty luciferase vector or BTG1 3’-UTR luciferase vector was transfected into 293 T cells that were seeded in 24-well plate 24 hours beforehand by using Lipofectamine 2000 (Invitrogen, Carlsbad, CA, USA). Forty-eight hours after transfection, the dual Luciferase Reporter Assay System (Promega, Madison, WI, USA) was used to measure luciferase activities. Each experiment was repeated three times and each measurement was taken in triplicate.

### RNA pull-down assays

Glioblastoma cells were seeded in 10 cm plate 24 hours before being transfected with desthiobiotin-labeled BTG1 3’UTR probe (Pierce RNA 3’ end desthiobiotinylation kit, cat# 20163). Forty-eight hours after transfection, cells were harvested and treated with cell lysis buffer containing 25 mM Tris-HCl pH 7.4, 150 mM NaCl, 1 mM EDTA, 1% NP-40, 5% glycerol. The RNA binding protein was enriched by using Pierce™ Magnetic RNA-Protein Pull-Down Kit (ThermoFisher, USA. cat# 20164) according to the manufacturer’s instruction. PUM2 was then identified by using anti-human PUM2 antibody in western blotting.

### RNA immunoprecipitation

RNA immunoprecipitation was performed as described in (Naudin *et al.*, 2017). Dynabeads ProteinG (Life Technologies) saturated with PUM2 antibody were used to pull down PUM2-RNA complex. The pull-down products were treated with DNase I (DNase I amplification grade, 18068-015; ThermoFisher-Scientific) before RNAs were segregated by using TriPURE (Roche). The relative quantity of BTG1 3’UTR RNA was evaluated using RT-qPCR as described previously.

### Statistics

All data are presented as the means±SEM as indicated in graphs. Student’s T test was used for comparison between two groups. All statistical tests were evaluated at the 5% significance level.

## Results

### PUM2 expression is elevated in glioblastoma tumor tissues and cell lines

**Fig. 1:**
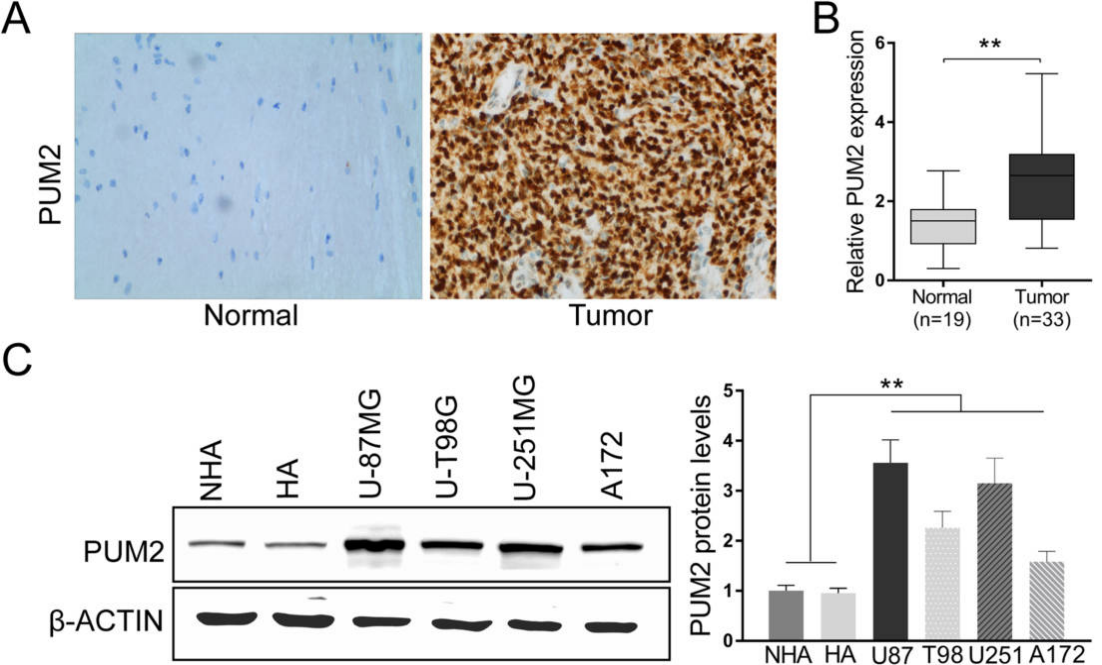
PUM2 expression is elevated in glioblastoma tumor tissues and glioblastoma cell lines. (A) Immunohistochemistry analysis of PUM2 protein expression in human glioblastoma tumor tissues and adjacent normal tissues using anti-human PUM2 antibodies. (B) RT-qPCR analysis of human PUM2 transcript in human glioblastoma tumor tissues (n =33) and adjacent normal tissues (n=19). Results are normalized to GAPDH expression. (C) Immunoblot analysis of PUM2 protein in indicated cell lines. Beta-actin was used as loading control. **p<0.01, Student’s t-test.

Glioblastoma is one of the most aggressive malignant tumors in CNS with rapid proliferation and extensive invasion into the adjacent brain tissues. However, the molecular mechanism underlying the malignant properties of glioblastoma remains poorly understood. PUM2 is known to be a positive regulator of cell proliferation. To investigate whether PUM2 contributes to the malignant properties of glioblastoma, we first checked the expression level of PUM2 in both glioblastoma tumor tissues and cell lines. As indicated in Fig. 1A and B, glioblastoma tumor tissues had elevated PUM2 transcript (Fig. 1B) as well as protein expression (Fig. 1A) compared to the adjacent normal tissues. Based on these findings, we suspected PUM2 expression would be elevated in established glioblastoma cell lines. We then tested PUM2 protein expression in multiple glioblastoma cell lines, including U-87MG, U-T98G, U-251MG and A172. As expected, in comparison with non-glioblastoma NHA and HA cell lines, PUM2 expression level is 1.58 to 3.56 times higher in glioblastoma cells than control cells (Fig. 1C). Therefore, our results suggest that PUM2 probably contribute to the malignant properties of glioblastoma.

### PUM2 is required for glioblastoma cell proliferation and migration

**Fig. 2:**
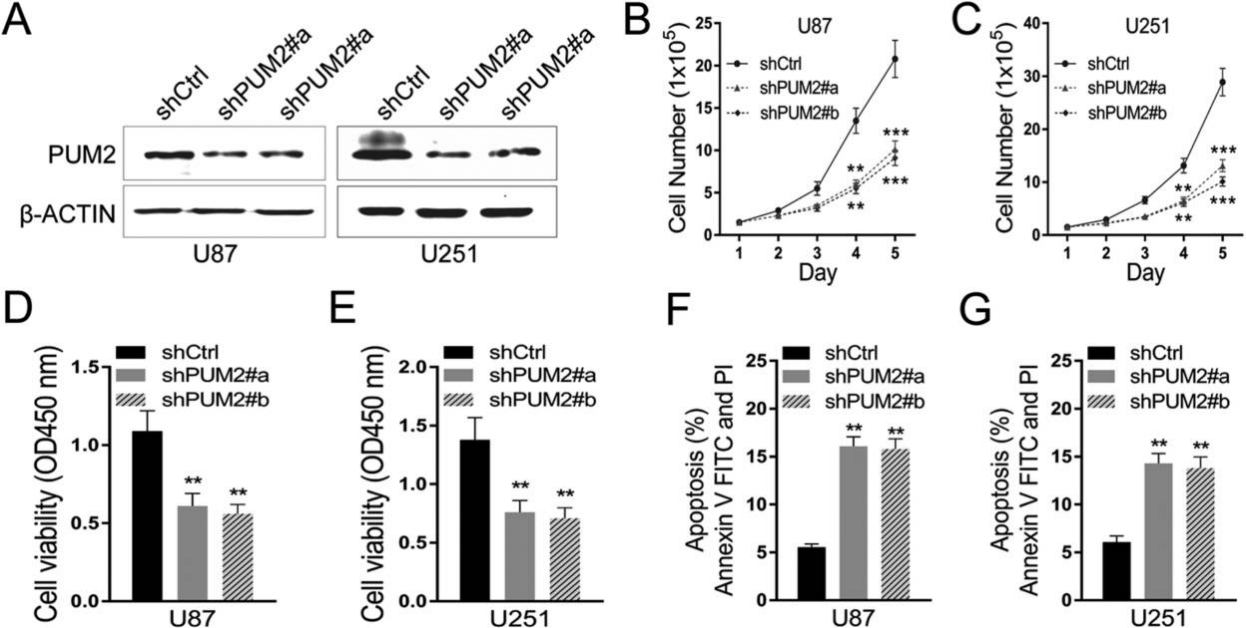
PUM2 is required for glioblastoma cell proliferation and viability. (A) Two shRNAs targeting PUM2 transcript effectively knock down PUM2 protein expression in U87 cells (left panel) and in U251 cells (right panel). Anti-human PUM2 antibodies was used for Immunoblotting. Beta-actin was used as loading control. ShRNA targeting GFP was used as a control. (B and C) PUM2 knockdown reduces glioblastoma cell proliferation. Cells were suspended and seeded at 1.5×10^5^/well in 6-well plates. Cells were counted manually every 24 hours. (D and E) Knockdown of PUM2 reduces glioblastoma cell viability. PUM2 knockdown glioblastoma cells were suspended and seeded at 1,000/well in 96-well plates.CCK-8 assay was used to evaluate cell viability 72 hours after seeding. For the details of screening stable cell lines after transduction with shPUM, see the text in the method section. Representative of flow cytometry analysis of (F) U87 and (G) U251 cells death after cells were deprived of FBS for 48 h. Data are presented as mean±S.D of three independent experiments. Each experiment had triplicate measurements (**p<0.01, ***p<0.001, Student’s t-test).

To further clarify PUM2’s role in the malignant behaviors of glioblastoma cells, we designed shRNA to knock down PUM2 expression in U-251MG and U-87MGcell lines. As indicated in Fig. 2A, two PUM2 shRNA (#a and #b) targeting different sequences of PUM2 transcript effectively knocked down PUM2 protein expression. Stable cell lines expressing PUM2 shRNA as well as control shRNA were then established for future experiments. The proliferating rates of stable lines were observed for a period of 5 days. Compared to control shRNA, PUM2 shRNA significantly reduced cell numbers over 5 days (Fig. 2B and C), indicating knocking down PUM2 suppressed glioblastoma proliferation. To test whether the slower proliferating rate is due to the decreased cell viability, CCK-8 assay was used to evaluate the viability. As indicated in Fig. 2D and E, knockdown PUM2 reduced the viability of glioblastoma cells by 50%. These results suggest that PUM2 positively regulate glioblastoma cell proliferation probably by increasing its viability. By using flow cytometry, we found that knockdown of PUM2 can significantly promote cell apoptosis in both U87 and U251 cell lines (Fig. 2F and G).

**Fig. 3:**
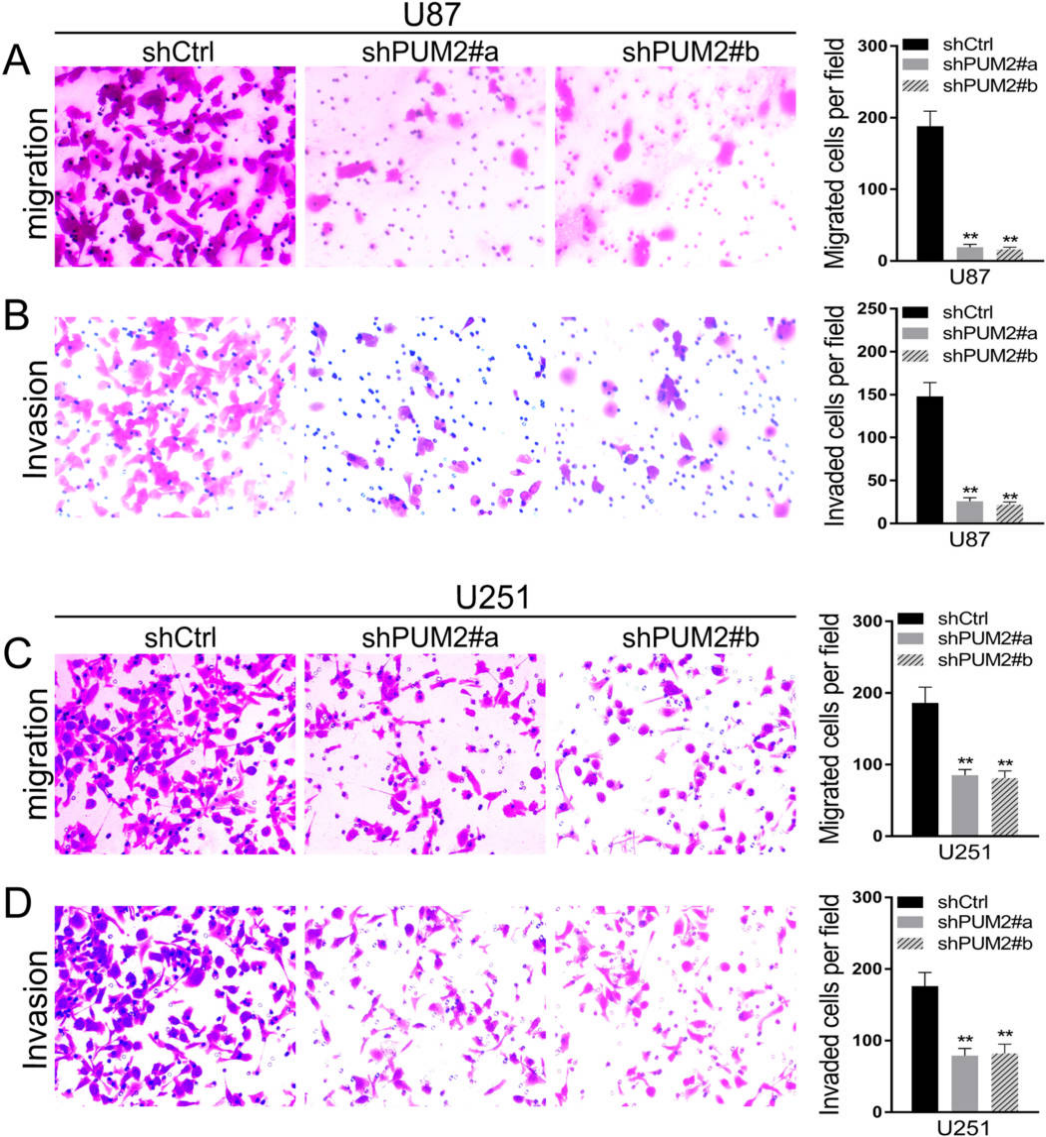
PUM2 is required for glioblastoma cell migration and invasion. (A and C) Knockdown of PUM2 suppresses glioblastoma cell migration. left panel: representative photograph of U87 (A) and U251 (C) cells that migrated through polycarbonate membranes. Right panel: statistical graph of the average number of cells in each group. (B and D) Knockdown of PUM2 suppresses glioblastoma cell invasion. Left panel: representative photograph of U87 (A) and U251 (C) cells that invaded through matrigel. Right panel: statistical graph of the average number of cells in each group. Cells were stained with crystal violet. Data are presented as mean±S.D. of three independent experiments. Each experiment had triplicate measurements (**p<0.01, Student’s t-test).

To figure out whether PUM2 could also contributes to the metastatic ability of glioblastoma cells, we further tested the migration and invasion capabilities in PUM2 knockdown glioblastoma cells. As indicated in Fig. 3A and C, PUM2 knockdown suppressed glioblastoma cell migration. Specifically, approximately 85% U87-MG and 50% U251-MG cells with PUM2 knockdown were blocked from migrating through the polycarbonate membranes. Furthermore, PUM2 knockdown blocked almost 80% of glioblastoma cells from invading through the matrigel (Fig. 3B and D). Thus, our results indicate that PUM2 promotes glioblastoma cell migration and invasion.

### PUM2 negatively regulates BTG1 expression via binding to its 3’UTR site

**Fig. 4:**
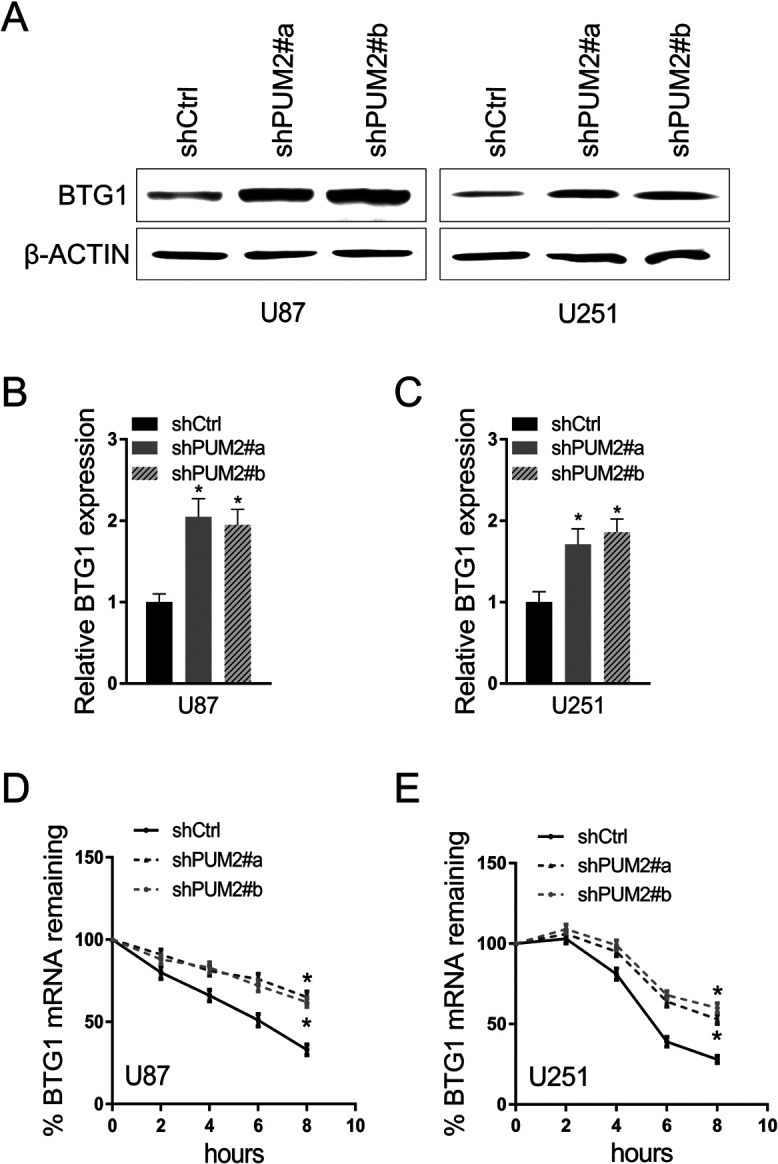
PUM2 represses BTG1 gene expression in glioblastoma cells. (A) BTG1 protein expression level is elevated in PUM2 knockdown glioblastoma U87 cells (right) and U251 cells (left). Human anti-BTG1 antibody was used for immunoblotting. Beta-actin was used as a loading control. (B and C) BTG1 mRNA is elevated in PUM2 knockdown glioblastoma U87 cells (B) and U251 cells. RT-qPCR was used to quantify BTG1 gene transcript. (D and E) Half life of BTG1 mRNA is elongated in PUM2 knockdown glioblastoma U87 cells (D) and U251 cells (E). Cells were treated with actinomycin D at 10 μg/ml at time 0. BTG1 mRNA were collected at the indicated time points after treatment and quantified using RT-qPCR. For RT-qPCR, GAPDH mRNA was used as a reference. For all PUM2 gene knockdown experiments, ShRNA targeting GFP transcript was used as a control. Data are presented as mean±S.D. of three independent experiment. Each experiment was measured in triplicate (*p<0.05, Student’s t-test).

**Fig. 5:**
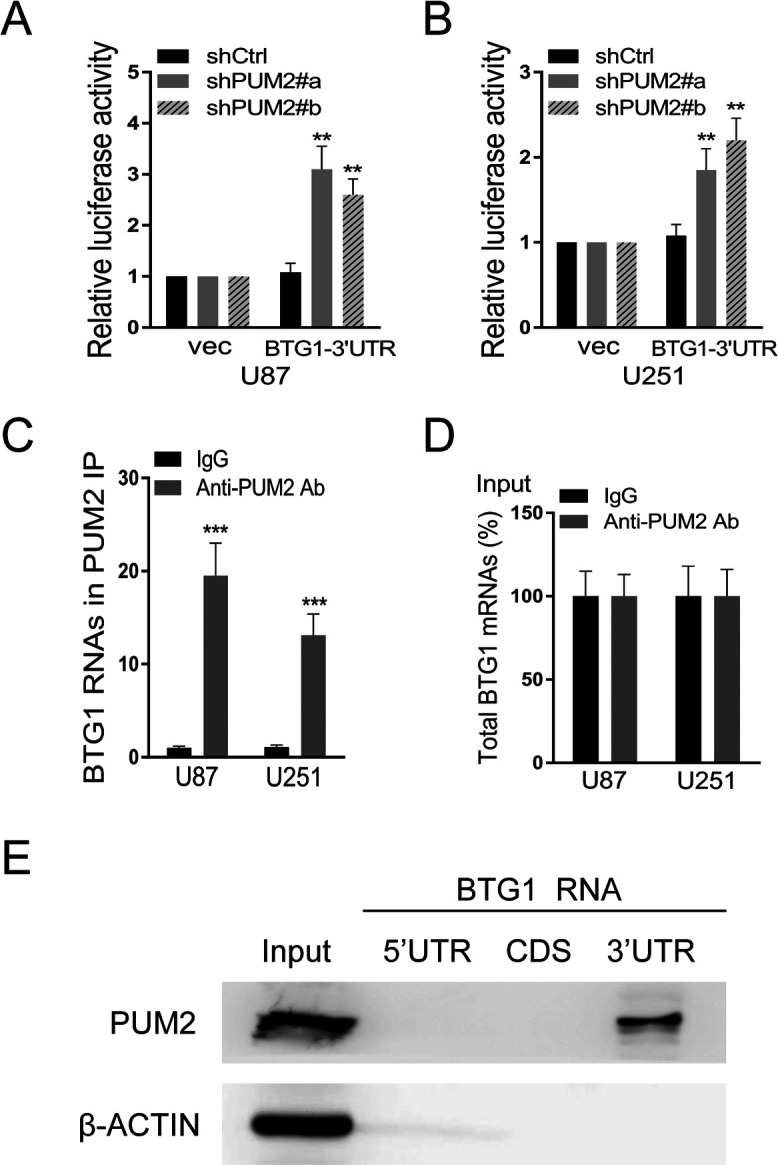
PUM2negatively regulates BTG1 gene transcription via binding to 3’UTR of BTG1. (A and B) Transcriptional activity analysis ofBTG1 3’UTR by using Dual-luciferase assays. Knockdown of PUM2 in glioblastoma U87 (A) and U251 cells (B) disinhibits BTG1 3’UTR. Renilla luciferase activity was normalized to firefly activity and presented as relative luciferase activity. (C and D) RNA immunoprecipitation assay. GAPDH was used as a control. (E) RNA pull-down assay. β-ACTIN was used as a control. As indicated in C–E, PUM2 directly binds to BTG1 3’UTR.Data are mean±S.D. of three independent experiment and each experiment was measured in triplicate (**p<0.01, ***p<0.001, Student’s t-test).

PUM2 is an RNA binding protein and functions as a translational repressor. It promotes stem cell proliferation via repressing cell cycle regulators’ expression. One of the well-known cell cycle regulators, BTG1, functions as a tumor suppressor by keeping cell cycle at G0 phase and its expression is down regulated in multiple malignant tumors. Here we hypothesize that PUM2 promote glioblastoma development probably by negatively regulating BTG1 expression. As indicated in Fig. 4A, knockdown of PUM2 led to increased BTG1 protein expression in glioblastoma cells. In addition, glioblastoma cells with PUM2 knockdown had one-fold higher BTG1 mRNA expression than the control cells (Fig. 4B and C). Furthermore, BTG1 mRNA in PUM2 knockdown cells had substantially prolonged half-life (Fig. 4D and E). To test whether the 3’UTR of BTG1 is the direct target of PUM2, we generated a luciferase construct with 3’UTR of BTG1 at the end of the luciferase coding sequence. As shown in Fig. 5A and B, luciferase activities were significantly higher in glioblastoma cells with PUM2 knockdown than in the control cells. To further clarify that PUM2 can directly bind to BTG1 3’UTR, we performed RNA immunoprecipitation assay and RNA pull-down assay. As indicated in Fig. 5C and D, BTG1 gene 3’TUR co-precipitated with PUM2 protein. In turn, PUM2 was successfully pulled down when using BTG1 gene 3’UTR as a bait (Fig. 5E). To conclude, our results indicate that PUM2 binds to BTG1 3’UTR directly and negatively regulates BTG1 expression probably by promoting BTG1 mRNA degradation.

### BTG1 promotes glioblastoma cell proliferation, migration and Invasion

**Fig. 6:**
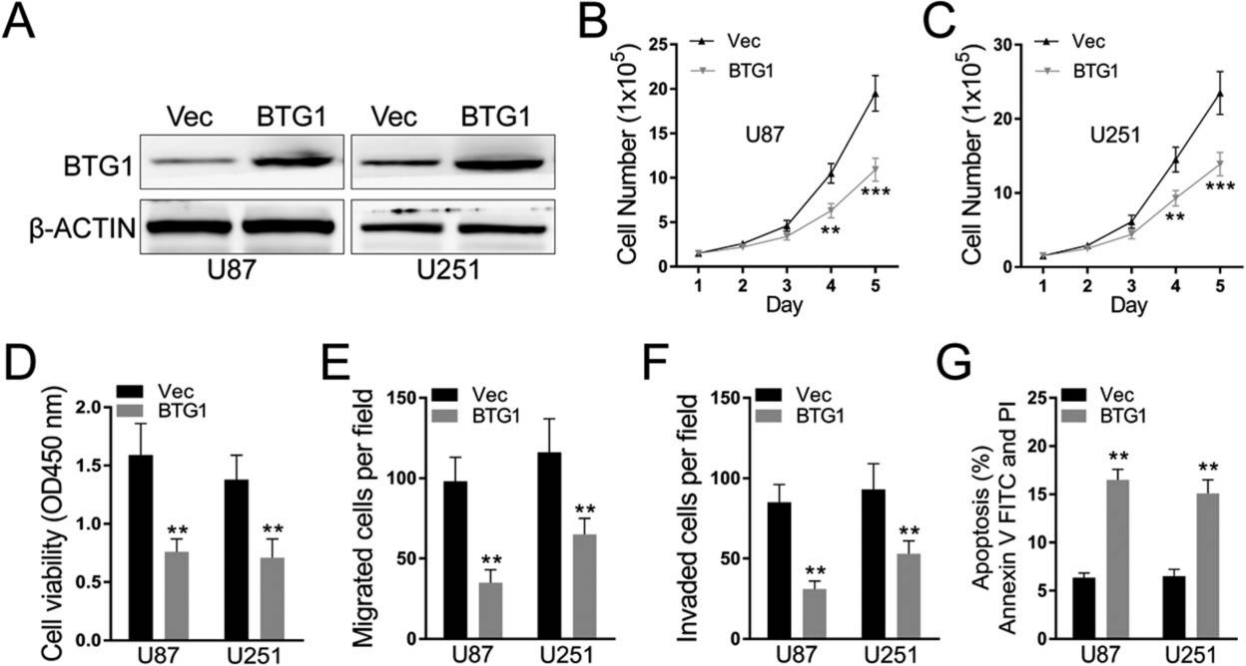
BTG1 inhibits glioblastoma cell proliferation and viability. (A) WB assay to detect protein expression levels. β-actin was used as loading control. (B) (C) Over-expression of BTG1 reduces glioblastoma cell proliferation in cell number assay. Cells were suspended and seeded at 1.5×10^5^/well in 6-well plates. Cells were counted manually every 24 hours. (D) Over-expression of BTG1 reduces glioblastoma cell proliferation in CCK-8 assay. BTG1 over-expression glioblastoma cells were suspended and seeded at 1,000/well in 96-well plates.CCK-8 assay was used to evaluate cell viability 72 hours after seeding. (E) Transwell migration assay and (F) trans well invasion assay to detect the cell viability. (G) Representative of flow cytometry analysis of glioblastoma cells death after cells were deprived of FBS for 48 h. Data are presented as mean±S.D of three independent experiments. Each experiment had triplicate measurements (**p<0.01, ***p<0.001, Student’s t-test).

To clarify BTG1’s role in the malignant behaviors of glioblastoma cells, we overexpressed BTG1 in U-251MG and U-87MG cell lines. As indicated in Fig. 6A, the expression level of BTG1 in the modified group was significantly higher than that in the control group. Stable cell lines with over-expressing BTG1 as well as control vector cell lines were established and used for future experiments. Compared to control, over-expression of BTG1 significantly reduced cell proliferation by counting cell number (Fig. 6B and C) and using CCK-8 assay (Fig. 6D), indicating over-expression of BTG1 suppressed glioblastoma proliferation. To figure out whether BTG1 also contributes to the function of glioblastoma cells, we further tested the migration and invasion capabilities in BTG1 over-expressing glioblastoma cells. As indicated in Fig. 6E and F, over-expression of BTG1 suppressed glioblastoma cell migration and invasion. Moreover, using flow cytometry, we also found that over-expression of BTG1 can significantly promote cell apoptosis (Fig. 6G). Together, these results indicate that BTG1 inhibits glioblastoma cell proliferation, migration and invasion.

### Knockdown of BTG1 restores the proliferating rate and migrating prosperities of glioblastoma cells with PUM2 knockdown

**Fig. 7:**
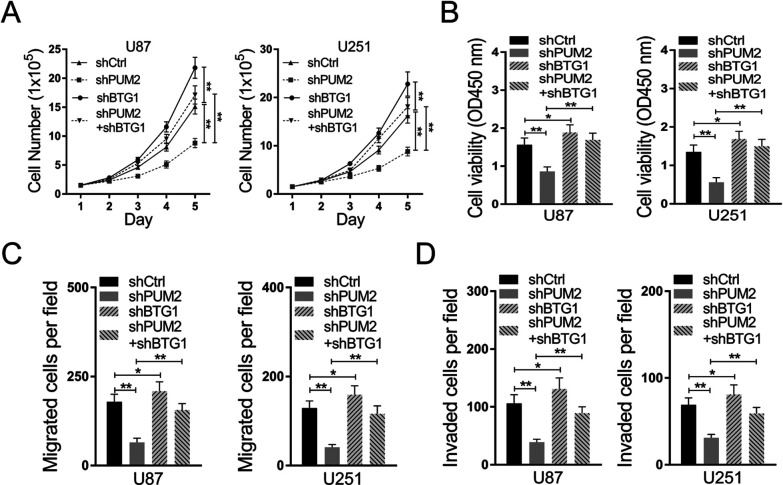
Effect of PUM2 knockdown on glioblastoma cell proliferation and migration is reversed byBTG1knockdown. (A) BTG1 knockdown reverses effect ofPUM2 knockdown on cell proliferation. (B) BTG1 knockdown reverses effect of PUM2 knockdown on cell viability. (C) BTG1 knockdown reverses effect of PUM2 knockdown on cell migration. (D) BTG1 knockdown reverses effect of PUM2 knockdown on invasion. Data are mean±S.D. of three independent experiments and each experiment was measured in triplicate. (**p<0.01, Student’s t-test).

Our previous results have shown that glioblastoma cells show substantially elevated PUM2 protein expression and knockdown of PUM2 suppresses glioblastoma cell proliferation and migration. Furthermore, PUM2 negatively regulates BTG1 expression in glioblastoma cells. Taken together, our findings suggest that glioblastoma cells acquire their malignant properties probably due to down regulation of BTG1 by PUM2. To test whether BTG1 is the critical mediator of PUM2 on glioblastoma development, we knocked down BTG1 in glioblastoma cells with PUM2 knocked down. As shown in Fig. 7A and 7B, knockdown of BTG1 completely got rid of the effect of PUM2 knockdown on cell proliferation and cell viabilities. In addition, knockdown of BTG1 restored the capabilities of migration and invasion in glioblastoma cells with PUM2 knockdown (Fig. 7C and 7D). Therefore, our results indicate that BTG1 is the critical target of PUM2 and its down regulation by PUM2 contributes to glioblastoma development.

## Discussion

Glioblastoma is one of the most devastating malignancies with treatment options far away from satisfactory. Despite of adjuvant treatment with temozolomide approved decade ago, the median survival remains as short as 18 months. In the meantime, advances in the pharmacotherapy is accompanied with substantial cost (Franceschi *et al.*, 2018; Hansen *et al.*, 2018; Monticelli *et al.*, 2018). Thus, uncovering the molecular genetics of glioblastoma is imperative for developing new treatment options with better cost-effectiveness. Recent studies focusing on the molecular targets that mediate the sensitivity and resistance to treatment have demonstrated that glioblastoma is molecularly heterogeneous. Multiple genes implicated in cell proliferation and differentiation, including the telomerase reverse transcriptase gene, c-MET, EGFR and NF1, have been showed to be mutated or overly expressed in glioblastoma (Appin and Brat, 2015; Bao *et al.*, 2014; Li *et al.*, 2016). In this study, we demonstrate that expression of PUM2, an RNA binding protein and positive regulator of cell proliferation, is elevated in glioblastoma tumor tissues and cell lines (Fig. 1A–C). Knockdown of PUM2 in glioblastoma cell lines suppresses tumor cell proliferation, migration and invasion (Fig. 2, Fig. 3). Interestingly, knockdown of PUM2 leads to increase of BTG1 expression in glioblastoma cells (Fig. 4, Fig. 5). In addition, knockdown of BTG1 reverses effects of PUM2 knockdown on tumor cell proliferation and migration (Fig. 7). Taken together, our findings indicate that elevated PUM2 expression and subsequent down regulation of BTG1 is one of the molecular characteristics of glioblastoma, suggesting that PUM2 and its downstream cellular pathways can be a potential pharmacotherapeutic target. Furthermore, monitoring PUM2 expression level can be potentially useful in evaluating treatment response.

Though roles of PUM2 in proliferation of germ cell and stem cell are well documented in the literatures, involvement of PUM2 in cancer growth remains controversial. Studies on bladder cancer development showed that up regulation of PUM2 is associated with inhibited cancer growth (Li *et al.*, 2018), while one recent study on leukemia showed that PUM2 sustains myeloid leukemia cell growth (Naudin *et al.*, 2017). As mentioned previously, PUM2 is broadly involved in regulation of many genes. Thus, the simple explanation of these controversial findings is that the net effect on cell growth resulted from posttranscriptional regulation by PUM2 is transcription spectrum dependent. In the glioblastoma cells, as demonstrated in our study, the net effect is suppression of cell growth which is probably due to down regulation of tumor suppressor genes such as BTG1 (Fig. 4). Consistent with its RNA binding prosperity, our results indicate that PUM2 can directly bind to the 3’UTR region and represses translation of BTG1 transcript. Furthermore, our results shown binding of PUM2 to the 3’UTR region can also affect the stability of BTG1 mRNA which partially contributes to the down regulation of BTG1 expression (Fig. 5). Therefore, our results suggest that the molecular mechanisms underlying posttranscriptional regulation by PUM2 are not limited to direct blocking of translational complex. Our results are consistent with the previously reported findings that demonstrate PUM2 reduces the stability of DUSP6 mRNA in tumor cells (Bermudez *et al.*, 2011).

BTG1 is a well-known tumor suppressor gene which functions through inducing G0/G1 arresting during cell cycle and promoting cell apoptosis. Its down regulation has been reported to be associated with tumorigenesis of multiple non-CNS cancers including bladder cancer, colon cancer, breast cancer and gastric cancer (Jiang *et al.*, 2017; Nan *et al.*, 2017; Zheng *et al.*, 2015; Zhu *et al.*, 2015). Evidences have shown that over expression of BTG1 can suppress cancer cell proliferation as well as migration and invasion. However, regulation of BTG1 in cancers of CNS remains unexplored. Here we demonstrate that down regulation of BTG contributes to the pathogeneses and aggressive behaviors of glioblastoma cells which is consistent with BTG’s roles in the pathogenesis of most cancers. More importantly, we identify PUM2 as one of the key regulators of BTG1 expression since knockdown of PUM2 in glioblastoma cells gives rise to increased expression of BTG1. Interestingly, knockdown of BTG1 not only completely reverses the effect of PUM2 knockdown but seems to over restore the capability of proliferation in glioblastoma cell (Fig. 6). This finding suggests that the extend of BTG1 down regulation is closely associated with the higher grade of glioblastoma.

Our results indicate that activities of PUM2 are critical molecular characteristics of glioblastoma cells and BTG1 is involved in PUM2 downstream cellular pathways, suggesting PUM2 and BTG1 can be the potential pharmacotherapeutic targets in treatment of glioblastoma.

## Disclosure of potential conflicts of interest

Yuanyu Wang, Weili Sun, Jiankai Yang, Liang Yang, Chen Li, Hongjiang Liu, Xiaopeng Liu, and Baohua Jiao declare that they have no conflict of interest.

## Funding

Not applicable.
